# Role of Immune Checkpoint Inhibitors in Advanced or Recurrent Endometrial Cancer

**DOI:** 10.7759/cureus.2521

**Published:** 2018-04-23

**Authors:** Ena Arora, Muhammad Masab, Priyanka Mittar, Vishal Jindal, Sorab Gupta, Claudia Dourado

**Affiliations:** 1 Obstetrics and Gynecology, Civil Hospital Chandigarh, Chandigarh, IND; 2 Internal Medicine, Albert Einstein Medical Center , New York, USA; 3 Hematology and Oncology, Albert Einstein Medical Center, New York, USA; 4 Internal Medicine, St. Vincent Hospital Worcester, Worcester, USA; 5 Hematology and Oncology, Albert Einstein Medical Center , New York, USA

**Keywords:** endometrial cancer, pd-1 inhibitor, checkpoint inhibition, immunotherapy

## Abstract

Currently, treatment options for patients with advanced or recurrent endometrial cancer remain limited. The current standard of care treatment for advanced endometrial carcinoma is a platinum doublet chemotherapy. Second-line treatment options overall are very limited. There is no optimal treatment option for patients who show disease progression with first-line therapy. Therefore, novel and more efficacious therapies for patients with advanced or recurrent disease are needed. Immune checkpoint inhibitors have demonstrated a very impressive safety profile and anti-tumor activity in patients with programmed death-ligand 1 (PD-L1) positive endometrial cancer who were pre-treated with chemotherapy. We have done a detailed review of the literature to emphasize the role of immune checkpoint inhibitors in the treatment of metastatic or recurrent endometrial cancer.

## Introduction and background

Endometrial cancer is the most common malignancy of the female genital tract in the United States. It was estimated that approximately 61,380 new cases of uterine cancer will occur in 2017, with 10,920 deaths resulting from the disease [1]. The main risk factors associated with developing endometrial cancer include increased estrogen exposure, complex atypical hyperplasia, tamoxifen use, Lynch syndrome, and diabetes mellitus. The incidence of endometrial cancer is increasing. This may be due to increased life expectancy and obesity.

Bokhman et al. postulate that there are two distinct pathogenetic types of endometrial cancer: type 1 and type 2 endometrial carcinoma. Type 1 disease accounts for 70%–80% of all endometrial cancers. These cancers are mainly of endometrioid histology. They are lower grade carcinomas that generally occur in younger women. Moreover, type 1 carcinoma is mediated by estrogen and has an increased percentage of mutations (including K-ras and phosphatase and tensin homolog [PTEN]) as well as defects in mismatch repair (MMR) genes resulting in microsatellite instability (MSI). Type 2 disease consists of higher grade adenocarcinomas and often non-endometrioid histologies. These cancers can show aneuploidy, p53 mutations, or overexpression of human epidermal growth factor receptor (HER-2)/neu [2]. 

The majority of endometrial cancers are caused by sporadic mutations. However, genetic mutations can cause endometrial cancer in about 5% of patients, occurring 10 to 20 years before sporadic cancer. In women with Lynch syndrome, the risk of endometrial cancer is 60%-70%. Lynch syndrome is caused by germline mutation in one of the many MMR genes (e.g., MLH1, MSH2, MSH6, and PMS2). MMR proteins maintain the integrity of the genome by rectifying base substitution mismatches and insertion-deletion mismatches resulting from DNA (deoxyribonucleic acid) replication errors. Mutations in MMR genes lead to a number of variations in microsatellite regions, causing MSI. The MSI affects genetic expression, resulting in aberrant cell growth or cell death [2-3]. Screening for genetic mutations (e.g., Lynch syndrome/hereditary non-polyposis colorectal cancer) should be considered standard of care in all patients with endometrial cancer but especially in those younger than 50 years of age [4-5]. 

Cancer immunotherapy is a new rapidly advancing component of cancer therapy, joining surgery, cytotoxic chemotherapy, radiation, and targeted therapy. The type of immunotherapy that has shown the most promising results involves antibodies to inhibitory immune checkpoint molecules. As a brief background overview, the human immune system is comprised of two arms: the innate and adaptive. The innate immune system includes dendritic cells, natural killer cells (NK), macrophages, neutrophils, eosinophils, basophils, and mast cells. The components of innate immunity do not depend on prior stimulation by antigens. The adaptive immune system includes B lymphocytes, cluster of differentiation 4 (CD4+) helper T lymphocytes, and cluster of differentiation 8 (CD8+) cytotoxic T lymphocytes, and requires prior antigen exposure mediated by antigen-presenting cells (APCs) for its activation [6]. 

Each human cell experiences multiple DNA damaging events daily [7], which are normally repaired by specific DNA repair pathways. However, there are some cells that are not repaired, and they eventually develop a potential to become malignant. They are killed by the tumor immunosurveillance system. Since a malignant cell can have multiple genomic mutations, many new tumor-associated antigens (TAAs) can get expressed [8]. These antigens are presented on tumor cell surfaces with their major histocompatibility complex (MHC) molecules. The next step is the recognition of an antigen-MHC complex by a T-cell antigen receptor. This step does require additional costimulatory signals that are provided by the interaction of cluster of differentiation 28 (CD28) receptor on the T-cell surface with B7 ligand molecules on the antigen presenting cells (APCs). This CD28 receptor/B7 ligand interaction stimulates the proliferation and function of T-cells. However, some malignant cells are able to evade the tumor immunosurveillance system by changing their own features as well as by modifying the tumor microenvironment. These evasive mechanisms represent the major area of interest in cancer immunotherapy.

Cancer cells can successfully evade the immune system by expressing fewer antigens on their surfaces or by down regulating the MHC class I expression [9]. They can also defend themselves from T-cell attack by expressing immune checkpoint molecules. These molecules are upregulated by cytokines produced by activated T-cells and are part of a normal negative feedback loop to control excessive tissue damage from inflammation by downregulating or suppressing T-cells [10]. Some of the receptor/ligand combinations between T-cells and tumor cells are inhibitory in nature such as PD-1/PD-L1 (programmed cell death receptor and ligand) and CTLA-4/B7 (cytotoxic T lymphocyte-antigen 4) [11-14]. CTLA4 counteracts the activity of the T cell costimulatory receptor CD28 for binding to the B7 ligand expressed on tumor cells and, therefore, CTLA4 functions as a negative immune regulator. Similarly, the linkage between programmed cell death protein 1 (PD-1) on T cells and the PD-L1 on the tumor cell surface inhibits T-cell proliferation thus resulting in T cell function inhibition [15-18].

Monoclonal antibodies (mAbs) targeting PD-1 and PD-L1, as well as mAbs targeting CTLA-4, have been approved by the Food and Drug Administration (FDA) for several malignancies, such as melanoma, non-small cell lung cancer, renal cell cancer, urothelial cancer, and lymphoma [19-21]. These are called immune checkpoint inhibitors (ICPIs). At this time, immune checkpoint inhibitors have been evaluated in endometrial cancers in the advanced disease and palliative treatment setting. 

## Review

Currently, treatment options for patients with recurrent endometrial cancer remain limited. The backbone of first-line palliative treatment for advanced endometroid carcinoma is a platinum doublet, usually consisting of carboplatin and paclitaxel. Triplet therapy with paclitaxel, cisplatin, and doxorubicin (TAP) has produced a response rate of around 60% [22]. However, triplet therapy is associated with significant toxicity. Generally, a doublet of paclitaxel and carboplatin (TC) is preferred due to improved tolerability and toxicity. There was a phase III non-inferiority trial comparing TAP and TC showing no significant overall survival difference [23].

Second-line cytotoxic options overall are very limited. There have been no optimal treatment options for patients who show disease progression with first-line therapy. Therefore, novel and more efficacious therapies for patients with advanced or recurrent disease are needed. PD-1 is an immune checkpoint receptor that has acquired special interest as a therapeutic target for various cancers because of its significant role in tumor immune evasion as described above. 

The interaction of the PD-L1 with PD-1 receptors can lead to the functional inactivation of T cells that allows the tumor cells to save themselves from T cell mediated tumor destruction. In this modern era of cancer treatment, monoclonal antibodies directed against PD-1 or PD-L1 can result in promising tumor responses, and many of these antibodies have been approved by the FDA for a wide spectrum of advanced/metastatic cancers. Vanderstraeten et al. did an immunohistochemical analysis demonstrating that 83% of primary endometrial tumors and 100% of metastatic endometrial tumors express PD-L1 [24]. Thus, the role of PD-1 and PD-L1 blockade in advanced endometrial cancers can be significant, and there have been some phase 1 and phase 2 studies done to evaluate the anti-tumor efficacy, safety, and survival analysis in this population. 

KEYNOTE-028 (NCT02054806) was a phase 1 open-label trial that was designed to evaluate the safety and efficacy of pembrolizumab, an anti PD-1 monoclonal antibody, in patients with PD-L1 positive advanced solid tumors. This study also included a subset of patients with PD-L1 positive endometrial cancer who had disease progression after standard therapy [25]. Patients received pembrolizumab 10 mg/kg every two weeks for up to 24 months. Primary efficacy endpoint was objective response rate (ORR). Secondary endpoints in this study were drug safety, duration of response, progression-free survival (PFS), and overall survival (OS). Of the 75 patients screened, 36 (48.0%) had PD-L1 positive tumors, and 24 patients (32.0%) were enrolled. Twenty-three patients were included in the efficacy analysis. The ORR was 13.0% (95% CI, 2.8% to 33.6%). Three patients showed confirmed partial responses. Stable disease was observed in three patients (13.0%), and 13 (56.5%) patients had experienced progressive disease. The median duration of stable disease was 24.6 weeks. This study did an efficacy analysis involving 23 patients. The median PFS was 1.8 months (95% CI, 1.6 to 2.7 months), and the six and twelve month PFS rates were 19.0% and 14.3%, respectively. The median OS was not reached, and the six and twelve month OS rates were 67.0% and 51.0%, respectively. Nineteen patients had their tumors sampled for MSI status. Of these, one patient (5.3%) had MSI-high status, and 18 patients (94.7%) had non MSI-high status. Of note, the only patient who was classified as MSI-high showed the best response [25]. This is important to highlight, because MSI-high status correlates with a high tumor mutational burden and much better therapeutic response. 

Multiple genomic studies have evaluated the degree of microsatellite instability. Tumors acquire MSI due to inherited germline mutations of mismatch repair (MMR) genes or the epigenetic inactivation of these genes. Tumors with a high load of MSI are called MSI-high (MSI-H). It is known that MSI-H causes a build up of somatic mutations in tumor cells and leads to a spectrum of molecular and biological changes including high tumor mutational burden, increased expression of neoantigens, and abundant tumor-infiltrating lymphocytes.These changes have been linked to increased sensitivity to checkpoint inhibitor drugs.

Recent reports of responses with PD-1 inhibition in endometrial cancers with high mutation rates suggest that anti-tumor activity with PD-1 monotherapy may be mainly driven by preexisting neoantigen reactive T cells. In a single-arm phase 2 trial, Le et al. looked at the role of evaluating PD L1 blockades in tumors that are mismatch repair–deficient (dMMR). A non–colorectal cancer cohort was reported that included two patients with endometrial cancer. These patients experienced a clinical benefit, with the non–colorectal cohort experiencing a response rate of 71% [26]. However, there are some patients that are PD-1 positive but still show no response to anti PD-1 therapy. Mehnert et al. did a case study to study the role of genomic mutations in patients with PD-1 positive endometrial cancer. They identified a single patient that showed rapid clinical improvement once pembrolizumab treatment was initiated, exhibiting a partial response after eight weeks and sustaining the response for more than 14 months [27]. Genomic profiling revealed that the patient had a DNA polymerase epsilon (POLE) mutation [27]. These exceptional results support the theory that the presence of POLE mutations may aid in identifying patients for whom pembrolizumab may be particularly effective, a concept that should be investigated further. 

## Conclusions

In summary, checkpoint inhibitors illustrate a very favorable safety profile and strong anti-tumor activity in patients with PD-L1 positive endometrial cancers who were pre-treated with chemotherapy. The FDA approved pembrolizumab in 2017 for the treatment of metastatic or unresectable metastatic MSI-H tumors that have progressed following prior treatment. This emphasized the role of MSI testing in endometrial cancer since it can have a strong impact on therapeutic options. 
